# When abortion is not within reach: Ethiopian university students struggling with unintended pregnancies

**DOI:** 10.1186/s12939-019-0925-2

**Published:** 2019-01-28

**Authors:** Mulumebet Zenebe, Haldis Haukanes

**Affiliations:** 1 Centre for Gender studies, College of Development Studies, University of Addis Ababa, P.O.Box 1176, Addis Ababa, Ethiopia; 2 Department of Health Promotion and Development, University of Bergen, P.O box 7807, 5020 Bergen, Norway

**Keywords:** Sexual and reproductive health, Unintended pregnancy, Abortion, Gender inequalities, Shame, Silence

## Abstract

**Background:**

In spite of increasing international commitment to young people’s sexual and reproductive health, unintended pregnancies remain a major problem for young women worldwide. This article explores the issue of unintended pregnancies among Ethiopian university students and investigates narratives of students who carried their pregnancy to term. Ethiopia’s relatively permissive abortion law forms part of the backdrop for the exploration. We also consider how socio-cultural and religious norms surrounding female premarital sex/pregnancy, and gendered and urban-rural inequities, play a role in how students handle the challenge.

**Methods:**

The article is based on research conducted among students at Addis Ababa, Jimma and Mekelle Universities in Ethiopia between September 2016 and June 2017. Drawing on an interpretative, phenomenological approach to science and employing a qualitative methodology, the authors conducted in-depth interviews with 53 students and 24 selected staff at the three universities, and held two focus group discussions with students at Addis Ababa University.

**Results:**

The study findings show three possible scenarios for how students can deal with an unwanted pregnancy. The first is to have the pregnancy terminated secretly, and thereby avoid the stigma linked to premarital pregnancy. The second is to make a deliberate decision to keep the pregnancy and face the consequences to come. The third scenario is found in cases where the student seems paralyzed by feelings of shame, and where she ends up keeping the pregnancy due to her inability to act.

Students who end up carrying their pregnancy to term face many problems. Few support structures at the university are in place to cater for their needs. Moreover, family support is endangered by pregnancy, as it puts the student at risk of being ostracized from her family due to the shame she has imposed on them. Shame and silence are thus important social forces in these students’ lives, underpinned by gendered inequities and patriarchal norms.

**Conclusions:**

Along with rural-urban and gendered inequities, the article demonstrates how a shame-silence nexus forcefully operates in the lives of female students struggling with reproductive challenges, and the serious consequences a pregnancy may have for those who carry it to term.

## Background

Since the International Conference on Population and Development (ICDP) Cairo conference in 1994 there has been increasing international commitment to young people’s sexual and reproductive health and rights and we have seen an expansion of sex education and reproductive health services directed towards young people globally. However, the efforts are far from sufficient to provide young people with the means they need to make reproductive choices according to their wishes and their life situation. Unintended and unwanted pregnancies remain a major challenge for girls and young women worldwide [1–4], partly due to restrictive legislative environments which hinder access to safe abortion (4). Ethiopia, in contrast to many other African countries, has a relatively liberal abortion law (see below), but this is not enough to secure young women access to safe abortion services. Clandestine abortions are still common [5] and unintended pregnancy is one of the main reasons for girls to drop out of school [6]. The sparse research available from the student population in Ethiopia suggests that untimely/unwanted pregnancies and abortion-related problems are prevalent at Ethiopian universities ([6], see also [5]).

This article addresses the issue of unwanted premarital pregnancies among Ethiopian university students, in a situation characterized by lack of public support structures, gendered and rural-urban inequities and a moral landscape which sees premarital pregnancy as deeply inappropriate and shameful, but where abortion is simultaneously strongly condemned [5, 7, 8]. Premarital sex in general and premarital pregnancy in particular are, in many places, regarded as highly inappropriate (see for examples [9, 10]), and the unacceptability of premarital sex is found to be a major reason for unmarried girls to avoid seeking advice and using reproductive health services even if they are available [10]. The dominant reproductive discourse in Ethiopia, propagated both by religious institutions and within families, seems particularly powerful in its condemnation of premarital pregnancy [7, 11–13]. Coupled with a strong demand to control social behavior in public [14], and highly authoritarian parent-child-relations [15, 16] sexuality-related subjects are surrounded by silence and shame both in private and public spheres, leading young people to keep problems within these domains to themselves [14].

Studies indicate that new ideas about love and sexuality are spreading among young people due to urbanization and media influences, and that students are increasingly active in sexual relationships [14, 17, 18]. There is thus a huge tension between religious/moral norms communicated in the family and by authoritative institutions, and ‘modern’ norms pertaining to sexuality and reproductive health prevailing among students. This tension plays out differently for male and female students, in ways that powerfully demonstrate gendered inequities in particular ways. Male students are normally condoned or sometimes even applauded when transgressing the norm of abstinence, while this is far from the case among female students. Young women thus have to negotiate sexual practices in a situation where they are both expected to avoid sex and at the same time encouraged or pressurized by their male co-students to be involved in sexual relationships [16, 18–21]. Recent studies on sexual relations among university students in Ethiopia show that it is mostly male students who initiate sex and decide when and how to have sex [18, 19]. A study on men’s involvement in women’s decisions on abortion further shows that pressure from male partners played a key role in making young, unmarried women decide to terminate an unplanned pregnancy [22].

Another equity-related dimension relevant for our analysis concerns the position of rural as compared to urban students. With the expansion of higher education in Ethiopia, there has been an influx of young women and men travelling from the regions and the rural areas to the major cities to study. Research shows that Ethiopian students in general lack knowledge about sexual and reproductive health, including contraception (see for examples [17, 22]). However, this challenge is far more severe among students who have grown up in rural contexts than among those living in big cities. Many first-year rural students experience a shock when they encounter a student culture where the pressure to enter sexual relationships is huge, and, as shown in a recent study from Bahir Dar University, they are highly vulnerable to peer pressure and to exploitation from older students [23].

Our analysis builds on material collected for an explorative study about students’ decision-making related to sexual relationships, fertility control and abortion among students at three universities in Ethiopia. In this article, we investigate the narratives of students who for diverse reasons ended up carrying their pregnancy to term, the students’ ways of handling the situation and the circumstances that led them to keep the child. Meselu and colleagues [7, 8], in their rich qualitative study on young women who had their pregnancies terminated, point to the fact that there is a “double shame” underpinning the decision-making process of whether to terminate the pregnancy or not: One created by the fear of going against God’s will and the other generated by the experience of betraying the family and not following parents’ guidance. Closely related to this, is the tendency among the women to keep the problem to themselves, and not to involve other people in the decision-making process. Meselu and colleagues found that most of their study participants had kept silent about their situation, not discussing their pregnancy with anyone. They argue that this silence does not imply a lack of reflection or moral reasoning. Silence is rather a way of “managing the self” in the highly unfortunate situation that the girl finds herself in, guided both by the norm of keeping quiet about sexual matters, and by the norm of parental obedience [8]. Employing a particular cultural model of moral reasoning (“the two hearts”), the girls actively weigh different alternatives against one another, and conclude that abortion will be the best and morally most sound alternative as it will avoid upsetting parents and retain family pride [7, 8].

Building on Meselu and colleagues’ work, our article further explores contradictory moralities, shame and silence as social forces in young pregnant women’s lives. We demonstrate the agonies the pregnancy causes for individual girls, and the serious outcomes it has for their wellbeing and future prospects. Discussing the girls’ struggle to cope, we question the role of universities’ health/support systems and their capacity to handle students’ pregnancies. As is the case in many other contexts, sexual and reproductive health services at Ethiopian universities are directed at preventing pregnancies, and to some extent at facilitating access to abortion. From 2005 onwards Ethiopia’s criminal code permits termination of pregnancies under a wide range of circumstances (e.g. cases of rape, incest or minority) and on the word of the pregnant women, i.e. no evidence is needed to prove the circumstances [24]. Access to safe abortion should thus - in theory at least - be within range for those who are aware of, and explicitly seek, this option [23]. However, no services are established to help young students who do not manage to avoid getting pregnant, and who, for varying reasons we will return to, keep their pregnancies. Quite the contrary; they are left to themselves and in some cases even forced to terminate their studies due to their circumstances. Following Meselu and colleagues’ reasoning about the “double shame”, we argue that the severity of the girls’ situation cannot fully be understood unless we look into religious and cultural norms surrounding female sexuality, chastity and obedience; the immense impact these moral norms have on students’ ways of handling their pregnant state; and how the girls’ norm transgression influences their relationship with their families.

## Methodology

### Study context – The universities and their reproductive health programmes

The research on which this paper is based, is part of a broader study researching university students’ ways of negotiating with competing discourses of sexuality, fertility control and abortion in Ethiopia.^1^ The research took place in three universities in Ethiopia, namely Addis Ababa, Jimma and Mekelle Universities. The three universities were purposefully selected for the sake of achieving a level of variation: Addis Ababa is the oldest and largest university located in the capital, while Jimma and Mekelle are regional universities located in the southern and northern parts of the country, respectively. In terms of reproductive health services all three universities have clinics that provide advice in reproductive matters, including the provision of long-term and emergency contraceptives to students. Students who decide to have their pregnancy terminated may also, in certain cases, get assistance to have the abortion carried out. While Jimma University has a special clinic that provides abortion service to students, the university clinics at Addis Ababa and Mekelle Universities will refer students to clinics outside the campus.

The universities’ gender offices, moreover, provide support to students in the area of reproductive health, such as organizing awareness creation activities and implementing orientation programs on reproductive health services for new students at the beginning of the academic year. During the program, students are introduced to the reproductive health services provided at the campuses, including the provision of contraceptives and counseling services. Moreover, the gender offices of the universities organize training and awareness creation programs to help students make informed decisions on reproductive health matters, including assertiveness training for female students. As will be further discussed below, the information and sexual and reproductive health services offered to students are far from sufficient, both in the areas of pregnancy prevention and termination, and with regards to assisting students who carry their pregnancies to term.

### Data collection

The research is based on an interpretative, phenomenological approach to science [25], and a qualitative, explorative research design was used in the project. We conducted semi-structured, individual interviews with students and selected staff at the three universities. At Addis Ababa, a total of 13 staff and 30 student in-depth individual interviews took place, in Mekelle 5 staff and 12 student interviews and at Jimma University 6 staff and 9 student interviews. To enrich our understanding of the students’ perspectives, we triangulated the individual interviews with focus group discussions ([26]:444ff.) and conducted two focus group discussions (FGDs) with female students at Addis Ababa University; one with 9 students born and raised in Addis Ababa, and one with 11 students studying in Addis Ababa but originating in other regions. This latter group also included students from rural areas. Participants of the individual interviews and FGDs were recruited with the help of the gender office of the universities and through snowballing. Key informants were identified and recruited partly by the Addis-based researcher on our team, (M. Zenebe) and partly by the gender offices of the three universities. A main emphasis was given to Addis Ababa University, partly because it has the largest and most complex student population in Ethiopia, but also because field access was comparatively better in Addis than in the other two locations due to the above mentioned researcher's familiarity with this university. Different interview guides were developed for the three groups of interviewees, all centered around the same topics: sources of information on reproductive health issues and reproductive health services for students at the university; sexual relationships among students; gender issues with regards to fertility control; and pregnancy and pregnancy termination, including aspects of the abortion law from 2005. In addition, key informants and individuals who were members of students’ organisations were asked specific questions about their respective offices/organisations.

Criteria for inclusion in the student-based part of the study was that participants had to be registered as active students at one of the three universities involved. Students with different regional backgrounds and from different study disciplines were purposefully recruited to include a variety of categories and contexts. In terms of gender, the majority were female students – both rural and urban-, but male students were also included in the research (all the FDG participants were women, but 14 of the 51 individual students interviewed were male). Most of the students who participated in the interviews and focus group discussions were Orthodox Christians, but Muslims and Protestants were also represented. Leaders and members of students clubs and associations including religious groups were purposefully included in the research as these clubs and associations run a number of reproductive health related activities. The staff interviewed as key informants for the research were professionals who work in the areas of gender, sexuality and reproductive health, as well as other staff members including student counselors and the Dean of Students (the latter at Addis Ababa University only). The interviews and focus group discussions were conducted between September 2016 and June 2017. The first part of the Addis Ababa student interviews and the focus group discussion with Addis-born students were conducted jointly by the first and the second author and held in English, while all other interviews were conducted by the first author, in Amharic. With the exception of a few interviews, which were held in the office of our Addis-based researcher, all the interviews were conducted at the gender offices of the three Universities. The interviews lasted on average an hour, with 25 min as the very shortest interview to 1 h and 30 min as the longest. The process of interviewing started at Addis Ababa University (AAU) where the main body of data generation was done and continued until saturation. Expanding the research to Jimma and Mekelle Universities confirmed saturation, as few new themes emerged from the interviews with these students. An explanation for the lack of regional differences in our material may be that students at Ethiopian universities are recruited from the whole country; applicants to higher education in Ethiopia cannot themselves chose where to study but are assigned a place in one of the many universities in the country. The student population at each university is thus very mixed in terms of regional background.

During the first round of interviews in Addis Ababa, we realized that the topic of unwanted pregnancies stood out as a key theme in both student and staff narratives; we were told stories about pregnant students who our interviewees had themselves encountered, the lives of whom had made a strong impression on them. In trying to enhance our understanding of these cases, we attempted to recruit study participants who had carried out a pregnancy while being a student. Through the gender office of AAU two women were recruited who were willing to share their stories and they were interviewed by the first author. These interviews, which were conducted in a private room at the gender office, were open-ended and gave the women the opportunity to tell their stories uninterruptedly. Learning about these cases threw new light on the narratives told to us by “ordinary” students and staff, and deepened our understanding of the phenomenon of university students’ unwanted pregnancy significantly. Reflecting this process of interpretation and understanding, our article takes these two cases as a point of departure, and moves on to investigate the topic of premarital pregnancy and related themes as encountered in the narratives of other students and staff.

As concerns the interviews with the “ordinary” students, we found that most students were willing to and indeed interested in discussing most issues related to our inquiry. In consideration of the sensitivity of the topic, the first questions posed to the students were general ones while more sensitive and specific questions were kept for the last part of the interview. For example, questions about sources of information on sexuality and reproductive health were raised at the onset of the interview while questions about unwanted pregnancies and abortion were addressed during the last section. Some students openly shared their own experiences, but many of our participants tended not to talk about their own sexual activity and reproductive challenges, but rather provided concrete examples from fellow students and cases they had heard about. During the focus group discussions, the students were more open and reflective and engaged in vivid debates about the topics introduced.

### Data analysis

Thematic analysis was employed to identify, analyze and report themes within the data set. The first author transcribed all the data material and translated the interviews that were conducted in Amharic into English. Transcripts from the interviews and focus groups were shared between the first and second author and were analyzed along with memos and notes written during data collection. Coding took place by manually marking the segments of data with descriptive words or category names. Themes were defined and refined to identify the essence of what each category communicated. Under each theme, sub-themes were identified and grouped. Interpretations were made after patterns and regularities were noted.

### Ethical considerations

Following Riessman’s recommendation of modifying certain “Western” standards of research ethics when doing research in non-Western contexts, [27], written consent was not requested, as signing a paper tends to cause unease in the study area. In the Ethiopian context, signing a document carries a history of suspicion about intrusion by authoritative bodies, notably the government. However, all study participants were informed thoroughly about the focus of the study, and verbal consent was obtained before the interviews began. The study participants were also informed about their right to withdraw from the interviews at any point in time should they so wish.

To ensure anonymity, the researchers did not record the names of the study participants in the field notes or in the transcripts. To protect the privacy of the informants, all names of informants in this article are pseudonyms. The project was approved by the academic commission of the Centre for Gender Studies at Addis Ababa University, and by the Norwegian Centre for Research Data as a sub-component of the larger project ‘Competing discourses impacting girls’ and women’s rights: Fertility control and safe abortion in Ethiopia, Zambia and Tanzania’ (57089/3/00SIRH).

## Results

### Facing pregnancy: Strategies and consequences

#### Students’ experiences with pregnancy – Two cases

Our presentation of pregnancy cases among students starts by an examination of the narratives of two of our study participants who themselves had given birth not long before the first author interviewed them. In line with the problem statement of the article, we focus on the decision-making process about whether to keep the pregnancy or not, the support structures surrounding them in their handling of the pregnancy, and the implications of the childbirth for their lives and well-being.

##### Almaz

Almaz, a student from a rural area in southern Ethiopia, was raped in the campus area early one morning. She says that she was very frightened and confused after the incident, and told a friend about it. The friend took her to the clinic for a check-up where she was told that she was not pregnant:

My period came after a month. It was when I went to a clinic for the second time that I was told I was pregnant; maybe 3 or 4 months pregnant. I was shocked. I could not study. I contacted the gender office. Then I went to my family. I found out that my family was already informed about my pregnancy. I think students from my area had already sent the information to the community. When I went home my mother told me to leave the place immediately even before my father came home. She said the family didn’t want to have anything to do with me. I did not even spend a single night at my parents’ place. A neighbor gave me 200 Birr and I went to my sister’s place and spent the night there. I returned to the [university] campus the next day. My family members have never called me or looked for me until this very day. It was the gender office of the university that provided me with support. When I came back from my home town, the university was closed and there were no students present. The gender office arranged for me to stay in a dormitory, but I was not allowed to use the student cafeteria. There were days when I did not eat. The director of the gender office used to give me some money. Sometimes they asked me to leave the dormitory. I worked with the painters of the maintenance office for 20 days and was given some money. I was so depressed. I didn’t want to live. I wanted to die. I decided to take my life. They asked me if my family would call me to take me, but I said no. They even collected money for transport but I refused to take it, because I have nowhere to go. My parents clearly told me that they refused to accept me.

Then the gender office arranged for me to go to a “safe home” for victims of gender-based violence. It was a good place. There I gave birth to a baby girl. The child is now at an orphanage [a different place than the “safe home”]. She will stay there until I graduate. It is a temporary place for her to stay. I left the safe home 14 days after I gave birth. Recently I went to the orphanage to see the child. It is only one month and some days since I gave birth. I don’t know how I can take my child and raise her. What [job] will I get after graduation? I have no interest in raising her… I am a protestant. I used to go to church but not anymore. My mother has not yet called me. I know they have heard that I gave birth. They don’t know I was raped. They thought I got pregnant willingly. In our place, unwanted pregnancy is considered a taboo. I don’t want to go back to my parents.

##### Selam

Selam is a woman born and raised in Addis Ababa. The circumstances surrounding her getting pregnant are less clear than in the case of Almaz. Her narrative indicates that, although she knew the father of the child from before and sometimes refers to him as her “boyfriend”, the intercourse that led to the pregnancy was not voluntary.

There was a man who wanted to be with me but I was not ready. He used to push me to have a relationship with him. The guy used to follow me since I was in high school. He said he was in love with me and he forced me to have sex. I didn’t know I was pregnant. …... Two months after I got pregnant, I told the man about my situation and he said I should have an abortion. I refused because I thought I may not get a chance to be pregnant and have a child later on in life. Though not planned, I wanted to keep the child that God gave me. I asked God to make everything alright.

As I said earlier, when I told my boyfriend about the pregnancy, he told me to abort it. I said I would never do that. I had some information on fistula and other health problems related to abortion. I had no information about how to solve problems related to pregnancy. Since I had difficulties while growing up and later in life, I was strong. My Orthodox religion didn’t allow me to abort. I also said to myself, if I abort, I will not get the eternal life after the life of this world. It is a sin to kill a life that hasn't got the chance to see this world. My religion supported me to keep the child. I have seen the world, but the child has not seen it and he or she has a right to do so. I believe I should not hurt others. I have seen so many challenges in life. So I said I should not hurt an innocent child.

I also had another reason to keep my child. I have no family and I was lonely, so I wanted the child to fill this gap in my life. I said I have to have a child to support me and encourage me. I believed I could overcome the challenge.

Selam speaks about the financial challenges that she faced, but also explains that she got some help. Among other things, she was entitled to free medical care from the government because of her poverty status. Her relationship with her birth family is complicated; she suspects that she was adopted into the family and that is why she does not feel well-treated by or close to any of the family members. However, when she informed the family about the pregnancy, her father got involved, and he tried to sort out the situation:

God helped me a lot and I became strong. I told my sisters, those who grew up with me, about my challenges. I then talked to my father. We were not communicating before that. I showed him my first semester grade [transcript]. He said it was good. Then I told him about the pregnancy. He said it was ok. He asked, “*What does your boyfriend think about the pregnancy?*”. My father said that if my boyfriend wants me to abort, he would take him to court. “*But if he wants to keep the child, then we will formalize it so you would get married*” he said. I was happy with my father’s reactions and felt that God listened to my prayer. I was glad that my father was ok with the pregnancy. I convinced my boyfriend that my family would support me, but he did not want to marry me. I did not tell this to my parents. I just told my boyfriend that they expect us to get married.

Selam eventually married her boyfriend, and they started to live together with his parents, but her difficult situation was by no means resolved through the marriage:

My husband does not like the child. He asked me to throw her away. He did not like her from the first day of her life. From day one of our marriage, he mistreated me. He was afraid of my family, that was why he agreed to live with me. But he treated me so badly… He always blamed me for giving birth to the child. He says, “*It is you who brought this problem to us*”. He even told me many times to throw our baby away. He does not even realize that what he said could hurt my feelings badly.

Selam tells about the challenges she faced when she tried to continue her education. Having nowhere to place the child and also meeting resistance from the university management regarding re-entry to her studies, her prospects were not good. However, she has got some support from an NGO to start a small business and hopes this will enable her to earn some money. She does worry a lot about her situation, though:

My baby is suffering. I am breastfeeding her, but I am not sure what will happen when she starts to eat food. What will she eat? I am stressed. I even think of giving her to an NGO. I don’t want my baby to suffer. My husband said I should give her away to an NGO. But I don’t regret giving birth to her.

Selam refers to herself as a strong woman, and shows pride in the decision she made to keep the child, although she doubts that others will have the same strength as her.

I was asking myself what could happen to other women if they have a similar experience. Will they have the perseverance like me? Will they lose hope easily? I would imagine not all women fight persistently and I can see how much they would suffer.

#### Support structures for pregnant girls – Where are they?

Almaz and Selam share many similar experiences; the violence/pressure that led to the pregnancy, the fear and anxiety following the realization of their circumstances and the fear of their future prospects. Neither of them discusses in detail the option of abortion, but from their narratives we get to know what led them to keep their pregnancies. From here their stories emerge as different: While Almaz did not know that she was pregnant until it was too late to have an abortion, and she had no option than to continue the pregnancy and give birth, Selam made a conscious choice to keep the child based on her faith and on her fear of the medical injuries an abortion could cause. Selam moreover felt that she wanted a child to fill the “family gap” in her life.

Both Almaz and Selam report severe implications of their pregnancies and of having a child for their (future) lives. What support structures emerge as important in their narratives? Who helped them in their difficult situation? Almaz identifies the gender office as her most important source of support; they helped her find a place to sleep and arranged for her to give birth in a shelter. However, the support received was not regular but rather of an informal nature: The director of the office personally gave her money and arranged for her to have a temporary job. Although vital, this support has clearly not been sufficient to ensure her well-being and health. Selam does not talk about any support from university offices at all but she mentions free medical care from the government as an appreciated help, and also some support from an NGO to help her establish a business.

The lack of formal support structures for women who become pregnant was confirmed by many of the key informants at the university, who gave examples of girls they have personally assisted in similar situations:

She was 4 months pregnant when she came to the university. She gave birth during the first semester. After she gave birth, students rented a house for her and I was informed about her case. The house was very small at ..[location]. The university did not have a system to support such students, so I had to raise money from friends and colleagues…. I took bottles of water. I also took some clothes from my house and went to visit the student. The student had literally nothing in that house. She was holding the child and the child was without any clothes. She had no food or anything. (Key Informant 5, AAU)

Based on such experiences, the need to improve and formalize assistance for such students was recognized:

We need to institutionalize all the support. You cannot support everyone from your own pocket. The university must be student focused. The solution should be systemic. I cannot help everyone. I may not have anything to give... I cannot help everyone and if I try to do that, I am sure I will burn out. (Key informant 5, Addis Ababa)

What about family support? Returning to the cases of Selam and Almaz, their situations show some similarities as neither of them received any significant material support from natal kin; Almaz has been completely rejected by her family and is in a destitute situation. Her daughter has been placed in an orphanage and it is uncertain whether Almaz will ever be able to raise her. Selam has an irregular relationship with her natal family, but they do not support her financially or practically in terms of taking care of the child. She is living with the family of the child’s father, but still faces severe difficulties, including economically. The question of leaving the child with an NGO has been raised although, so far, she has done nothing to make this happen.

When it comes to assistance from the child’s father, this is of course irrelevant in Almaz’s case, but even Selam, who married the father of her child, is not supported by him, either financially or emotionally. According to other students, her case is not the only one; support from male partners seems to be the exception rather than the rule when a girl finds herself pregnant. As voiced by a male Addis-based student:

If a woman gets pregnant, the man would deny he is responsible for the pregnancy and would leave the woman. Her parents would kick her out of their house. What would she do? If the child is born, she would suffer. How could she raise the child? (Interviewee 3, male, AAU,)

Another student confirmed the lack of male involvement in pregnancy cases in general, but mentioned the rare possibility of the man’s family helping out, provided that he is seriously involved with the girl:

The male student would look for a solution if he loves his girlfriend but this is rare. Usually the men ignore the girl after she gets pregnant. It would be the girl’s responsibility. However, if he loves her, he would take her to his family (Interviewee 10, male, AAU)

#### Shame as a socio-cultural force in the lives of young women

The narratives of Selam and Almaz exemplify the hardships that young Ethiopian women who experience mistimed and socially unacceptable pregnancy are likely to face. Of the two, Almaz seems to have faced the greatest loss, being abandoned by her family because of the shame that she imposed upon them. Although her case may be an extreme one, the fear of parents’ reactions is recognized by many of our study participants as the key factor in making abortion stand out as the best and most likely option a female student would choose should an unwanted pregnancy occur. The reason given is precisely what caused Almaz’s parents to react in the way that they did: A daughter’s pre-marital pregnancy is shameful and may ruin family pride and reputation.

Many students, both male and female, pointed out how differently an unwanted pregnancy would strike gender-wise:

The girl’s family would be more upset than the boy’s family. It is about their pride. It is about their status. When their daughter gets married without any children [i.e. before having a child], they would be happy (Interviewee 10, male AAU).

A few spoke about the existence of a certain pressure from male partners to make their girlfriend choose the abortion alterative in case of pregnancy, similarly to what we saw in Selam’s case. More than pressure from partners, students voiced the fear of disappointing parents, who have invested so much in securing them a good education, as a main reason for choosing an abortion. This fear was often contrasted with the role played by religion in the decision-making process:

As one female student said:

When she [the university student] gets pregnant, she would have a moral dilemma. The family has high expectations. She would not consider her religion, but makes a decision to abort in order protect the name of her family. Before religion, you give priority to your pride. As for religion, you ask God’s forgiveness later. (Interviewee 8, female, AAU*)*

A male student confirmed this view, stressing the role of both community and family as more important than for a girl’s decision that her religious believes:

Religion has a big influence. Since a student thinks about her eternal life, it has influence. But I think the influence of the community and parents will win, they have more influence than religion. Due to this reason, a female student will abort if she gets pregnant (Interviewee 20, male AAU)

Another female student, who described herself as a very religious person, spoke warmly about her father and everything that he had done for her, revealing that she would never be able to let him down and come home pregnant:

As for me, I don’t talk to my father about sexual issues. He loves me. I know it is a big sin to have an abortion, but still I would go for it. I should consider what is best for my family. My father did not get the kind of education I have. He worked hard his whole life. He was a driver in the desert. So I don’t want to get pregnant and disappoint him. I cannot let him down. (Interviewee 2, female, AAU)

The preferred solution for a student who becomes pregnant would be, according to our study participants, to have the abortion carried out in secret and without the parents knowing anything about it. However, in our many interviews with students and staff we have encountered a number of pregnancy cases where the young women in question have remained silent about their condition, but where they have *not* acted to have an abortion. The girls encountered in many of these narratives appear to be overwhelmed by feelings which not only keep them silent but also unable to seek a solution:

A student in our college had a boyfriend who was a graduating student. She was a student at the English department. Her boyfriend had asked her to have a child with him before he graduated and left the university. She became pregnant but he left the campus and disappeared. The student tried to hide the pregnancy by not eating food and wearing tight clothes. She came to my office many times but was not able to tell me that she was pregnant. She is from […rural area in one of the regions] and was not able to speak to me in Amharic. She was only able to say, 'there is a problem' but was unable to describe what the problem was. She did not tell her friends that she was pregnant either as she was very ashamed. Finally, she collapsed in her dormitory. Her friends took her to a hospital and that was when her case was disclosed. (Key Informant 7, AAU)

A key informant at Jimma University shared a similar story. The student in question came to see our key informant in her capacity as health worker for the alleged reason of having parasites. The health worker suspected that it was more than parasites, and ordered an additional examination. The test result showed that, rather than parasites she had a positive pregnancy test:

The student couldn’t accept that she was pregnant. She said she was a virgin. I asked her if she had had sex with her boyfriend even just once but she said no. I also asked if she wore a man’s trouser and was in contact with sperm. Finally, she said she would take me to court for saying that she was pregnant, and left the room. It has been a month and half since this happened and she didn’t show up to see me again. One day, I met her friend here at the clinic, and the friend told me the student was pregnant. (Key informant 3, Jimma University).

Yet another case, told to us by a student, speaks about complete secrecy and denial:

There was a student who was pregnant. We asked her about her pregnancy but she denied she was pregnant. She did not tell anyone. But finally she entered her labor, and we called an ambulance and she gave birth. We visited her at the hospital and we collected money and gave her. Had she told us, we would have looked for a solution. (Interviewee 19, female, AAU).

Dramatic cases of students trying to hide pregnancies were presented to us at Mekelle University; these were cases of students who had left their newborn babies to die:

Shockingly, there was a girl who gave birth in the campus and put her baby in a sewage pipe. Unfortunately the baby was found dead after some days. The girl was taken to court and convicted of murder and sentenced for six years of imprisonment. (Key informant 6, Mekelle University)

A student in this campus [the same campus as above] gave birth at night and put her child on top of a roof. Vultures were trying to snatch the baby. The mother was found in the exam room. It was the university janitors who brought the child to us in the morning. The student said she got pregnant because she was raped on the street.. When students give birth at campus, we do take the babies and give them to organizations for adoption. But the girl I mentioned to you refused to give her baby away. After she got some psychological treatment, she took her baby to her family and she completed her studies this year. (Key informant 5, Mekelle University).

In the introduction we argued that rural students face more challenges than urban students as they are generally less informed about reproductive health matters, and too shy to ask for help when they face a problem. Rural-urban differences are acknowledged by a number of our study participants, key informants and students alike, both pertaining to levels of knowledge, assertiveness and how to deal with unwanted pregnancies. As expressed by a student during the focus group discussion:

Urban students are different from rural ones because they always ask for more information. They are not shy looking for a solution. They openly discuss the issue. Rural students are shy and hence can’t easily find a way out (FGD with female students from the regions, AAU)

An urban student formulated it even more directly:

There were some students who were pregnant. Those from Addis, they have an abortion. The rural ones don’t know where to go for help (Interviewee 7, female, AAU)

However, even if there is a “knowledge gap” between rural and urban students, and rural students tend to be even more powerfully governed by the “culture of silence”, the shame of improper and untimely pregnancy and the fear of disappointing ones family “who has high expectations”, was found to be widespread including among students from urban backgrounds.

## Discussion

Students at Ethiopian universities increasingly engage in sexual relationships, but some do not know how to protect themselves properly, or are victims or sexual assault, and find themselves facing an unwanted pregnancy. In such cases, three possible scenarios seem to emerge for how to deal with the situation: Firstly, there is an option of the “lesser shame” [28], namely to terminate the pregnancy secretly and thereby avoid the problems linked with untimely pregnancy and having a child out of wedlock. According to our study participants, this is an option preferred by the large majority, in spite of the religious condemnation of abortion. As mentioned above, the arguments the students present for choosing this option are very similar to those presented by the participants in Meselu et al.’s study. The fear of disappointing ones parents, and the shame involved in admitting premarital sex in front of the family, weigh more heavily than religious norms. After the new abortion law was implemented in 2005, legal abortion can be granted for a number of reasons. Even if many students lack detailed information about the law and its provisions [5], and young women continue to seek illegal services of abortion because of insufficient awareness [12] access to safe abortion services has improved substantially during the last decade, and this probably contributes to making termination the preferred alternative for students.

Religion is recognized as having very strong influence on the lives of Ethiopians and cultural norms related to sexuality are highly influenced by religion [29–31]. Not all students are thus able to act against their religious belief when faced with an unwanted pregnancy. In such situations, a second scenario emerges for how to deal with the problem, namely to decide to carry the pregnancy to term. Selam presents us with a highly relevant example of a student who has adhered to her faith when making her choice; she presents her wish to keep the child as religiously motivated, and is clearly proud of the choice that she has made.

The third scenario is manifested in the cases presented in the last section of our findings, that emerged so powerfully in our material; cases where the pregnancy is kept because of a *lack of ability* to act, and where overwhelming feelings of shame seem to play a key role in determining the outcome.

Let us recapitulate some of the consequences of keeping a pregnancy for female university students. Unlike the situation that is commonly found in some other African contexts (8, 9), Ethiopian learning institutions do not require students to abandon their studies because of pregnancy. However, in practice, it is very difficult for a pregnant girl to continue her studies. She may experience ridicule from other students; as we have seen there are cases where students try to hide their bodies or even stop eating properly to keep the pregnancy invisible and thereby avoid the shame and humiliation they may encounter in dormitories and classrooms. Even more problematic is the fact that few, if any, support structures are in place to take care of pregnant students, let alone single mothers [23]. The universities have no maternity leave system, no childcare services and no housing arrangements for students with children. Although Ethiopian universities have a cost-sharing system for the financing of higher education which among others allows students to take a loan from the government to cover housing and other expenses, economic support from the family is essential to be able to study. When faced with an unwanted pregnancy extra support from the family is needed to cover both the expenses related to the baby and any delay in studies that childbirth may cause. As we have seen in the cases of Almaz and Salam, obtaining such support from the family is not easy. Selam, who kept the pregnancy mainly for religious reasons, had a weak relationship with her birth family long before the pregnancy occurred. After the baby was born, her family did not provide her with any substantial material support but she received some degree of “moral” support from her father. Her weak relationship with the family may actually have been an additional factor in her decision not to go for abortion and to keep the baby. She may have felt that she did not need to protect her family from the shame of an unwanted pregnancy, and she does not seem to have feared disclosure to her father. His reactions to the pregnancy were not very negative, but, to secure her future, he did get involved in making marriage arrangements something which she – at least initially – was happy about.

Almaz’s case demonstrates how strongly socio-cultural norms surrounding female sexuality and chastity may operate in cases of unmarried women who end up pregnant. Without even being able to communicate to her family that the pregnancy was a result of a rape, Almaz has imposed a shame on the family which they feel so strongly about that they have turned their back on her completely.

The severity of Almaz’s case provides us with a strong backdrop for understanding the other cases presented above where feelings of shame, probably combined with or embedded in a strong fear of family reactions, seem to have completely muted the girls. Lack of knowledge about sexual and reproductive health, and of pregnancy may be one reason why the girls do not seek help; studies have shown that reproductive health information doesn’t reach out sufficiently to the student population, and to female students in particular [23]. However, in some of the cases presented in this article it is likely that the pregnancy has been kept for no reason other than shame and fear, a shock which paralyses the girls to the extent that they become unable to seek advice and services in time for a potential abortion. In contrast to the participants of Meselu and colleagues’ study [7, 8], who managed to keep the pregnancy hidden from their families until it was terminated and thus avoided the worst of the shame-scenarios, namely exposing the pregnancy to the public, the young students who carry their pregnancy to term and give birth to a child can hardly do so. They thereby have to face the risk of being ostracized from their families and left to fend for themselves, a scenario difficult to grasp in a context where the family as a social unit is of crucial importance for social and material survival [32].

How can we further understand the shame-silence scenario? As argued above, sociocultural and religious norms overlap in their strong condemnation of (female) premarital sex. A DHS-based study from 2014 [17] shows that reported rates of premarital sex are still very low in Ethiopia (around 10% in the DHS data from 2011), as are rates of premarital childbirth (1–2%). These numbers should be treated with caution; since the topic is highly sensitive, rates are likely to be underreported. Another factor playing into the picture, is low average marriage age; the earlier a girl marries, the more likely it is that age of marriage and age of first sex will overlap, and early marriage is still very common, particularly in rural Ethiopia. Urban women, and women with higher levels of education, are more likely both to postpone marriage and to have sex before marriage than rural and/or uneducated women [17]. Numbers are nevertheless low compared to most other African countries [33]. As discussed above, norms about male- female relationships are changing among students of today, who are far more exposed to media influences than previous generations [14] but overall societal norms are not changing at the same pace and parents still strongly expect their daughters to be married before entering a sexual relationship [32, 34]. Partly due to these conservative norms, but also caused by parents’ limited knowledge of sexual and reproductive health and the fear that discussion about sex would encourage premarital and thus inappropriate sexual activity, communication between parents and children on sexual and reproductive health matters is largely lacking [35]. This lack of openness, combined with authoritarian parenting norms and strongly hierarchical parent-child relations, increases the difficulty of disclosing an unwanted and highly shameful pregnancy to the family and to society in general.

An underlying dimension in all of the three scenarios, but weighing most heavily in the latter one, concerns gendered inequities and the way they unfold both in interactions between students and in society at large. In general, patriarchal attitudes are prevalent in Ethiopian society and women do not participate equally in the social, economic and political spheres of the country [32, 36]. Prevalent gender norms influence the way female students are expected to behave in their relationships with men; they are expected to be exacerbate shyness, reservation and submissiveness [6, 7]. Female students face the burden of protecting themselves during intercourse, but are also those who have to solve problems related to unwanted pregnancy. While male students, for various reasons including lack of financial resources and/or fear of their own future, seem inclined to run away from potential fatherhood responsibilities, female students risk having their lives ruined should they carry their pregnancy to term.

The cases of Almaz and Selam demonstrate such gendered inequities very clearly; they have both experienced the not uncommon scenario of sexual violence and have had to face the consequences of it in brutal ways. Selam was made to marry the perpetrator and is now suffering from living in a very difficult relationship. Almaz has felt the consequences of her (involuntary) norm transgression even more severely in that she was expelled from her family and even received threats of injury when she turned up pregnant in the village.

Rural-urban differences seem to play a role in determining which of the scenarios is most likely to unfold in each case, and the implications for the student in question. As explained above, rural students are vulnerable to exploitation by older mates but are also generally less informed about sexual and reproductive health issues and thus may face a greater risk of experiencing an unwanted pregnancy. Religious and sociocultural norms condemning premarital sex and childbirth are likely to be even stronger in rural than in urban areas. This may make it more difficult to bring home news about an untimely pregnancy for a rural student than for an urban one– something which the case of Almaz is an indication of.

A final reason why shame is felt so severely by some students, to the extent that they feel unable to speak, act and move, may also have to do precisely with their status as students. In the Ethiopian context, being admitted into a university is a great achievement in itself and raises expectations of success in many families, but also increases the disappointment of the family when the chance is spoiled by an unwanted pregnancy. Parents’ severe reactions to their daughters’ untimely pregnancy and childbirth may therefore not only be caused by the shame she has brought upon them, but also by the “failure” of their investment, and a reluctance to take on the burden of supporting yet another member of the family.

## Conclusions

In spite of an increased emphasis on sexual and reproductive health information and services at universities, and a relatively permissive abortion law which has expanded access to safe abortion for young unmarried women in Ethiopia substantially, many female university students are faced with pregnancy related problems. Our article has explored possible scenarios for dealing with untimely and unwanted pregnancies, focusing in particular on some cases where the pregnancy was carried to term. We have shown that the reasons for not seeking an abortion are manifold, ranging from not being able to detect the pregnancy in time, via firm religious beliefs, to paralyzing shame and/or denial of the situation. We have demonstrated the serious consequences that the pregnancy may have for those who end up giving birth to a child rather than having an abortion, and have argued that university support structures are, to a large extent, failing to take care of those who give birth while being enrolled as students. At the core of the problem are tensions between different norms and expectations concerning students’ sexual behavior, and underlying gendered inequities implied in these norms. Students increasingly see it as “modern” and “cool” to be involved in relationships, and there is considerable pressure on female students to have sexual relationships. At the same time societal norms, founded in religious beliefs and communicated in families, are still very strict when it comes to female premarital sex. Young unmarried women may experience condemnation when they transgress these norms, in particular when their norm transgression becomes evident to the public eye in the form of a growing belly. As we have seen, it is difficult for most students to bring home news about such an untimely pregnancy. Some are unable to do so for fear of the consequences, while others experience ostracization when the news are disclosed to their parents. The shame-silence nexus operates forcefully in the lives of many young female students. Strategies for improving sexual and reproductive health services at universities need to take this more seriously, paying attention not only to those who seek to prevent and terminate pregnancies, but also to those who have babies. Universities also need to strengthen their programmes for improving gender equality on campus, and to work not only to improve female students’ self-awareness and self-esteem, but also to transform cultures of masculinity and encourage sexual responsibility among male students.

## Footnotes

1 It is a Post-doctoral research project with the title *“University Students Negotiating Competing Discourses of Sexuality, Fertility Control and Abortion in Ethiopia”*. The study is part of a larger project entitled “*Competing Discourses Impacting Girls’ and Women’s Rights: Fertility Control and Safe Abortion in Ethiopia, Tanzania and Zambia*”, a three year project (2016–2018) financed by the Research Council of Norway (project number 249686), and affiliated to the Centre for Excellence scheme to the Centre for Intervention Science in Maternal and Child Health (CISMAC) at the University of Bergen, Norway
